# Eco-friendly reduced graphene oxide@potash alum-based composite membranes for efficient separation of dyes and selective removal of contaminants from wastewater

**DOI:** 10.1039/d6ra00858e

**Published:** 2026-07-02

**Authors:** Irsa Munwar, Akbar Ali, Ashique Hussain Jatoi, Khalid Hussain Thebo, Ahmed Nadeem

**Affiliations:** a National Centre of Excellence in Analytical Chemistry, University of Sindh Jamshoro Pakistan; b MIIT Key Laboratory of Critical Materials Technology for New Energy Conversion and Storage, State Key Laboratory of Urban Water Resource and Environment, School of Chemistry and Chemical Engineering, Harbin Institute of Technology Harbin 150001 PR China; c Department of Chemistry, Shaheed Benazir Bhutto University Shaheed Benazirabad Pakistan; d Department of Chemistry, Mirpur University of Science & Technology (MUST) Mirpur Pakistan khalidthebo@yahoo.com; e Department of Pharmacology and Toxicology, College of Pharmacy, King Saud University Riyadh 11451 Saudi Arabia

## Abstract

Graphene oxide (GO)-based membranes have gained significant attention for various separation applications over the last decade. However, achieving and maintaining the stability, high permeance, and high rejection of the membrane are still challenging tasks for the scientific community. In this study, we fabricated a reduced GO@potash alum-based (rGO@PA) composite membrane *via* a simple vacuum filtration method by modifying GO nanosheets with potash alum as the active material. The incorporation of a natural source of alum enhances the membrane stability and facilitates efficient stacking, while the high porosity and hydrophilic nature of the composite improve mass transfer, resulting in superior permeability. The as-prepared rGO@PA composite membrane achieves a high water permeance of up to 180 ± 10 L m^−2^ h^−1^ bar^−1^. Further, the rGO@PA composite membrane demonstrates excellent dye rejection for anionic dyes, *e.g.*, Congo Red, Eosin Y, and Rose Bengal, achieving rejections of up to 99.9%, 99.8%, and 99.2%, respectively. Besides these, the membrane also shows great rejection for cationic dyes, such as 99.0%, 99.6%, and 99.2% for methylene blue, methyl green, and methyl violet, respectively. In addition, the as-prepared membrane maintains its performance for over 15 days under aqueous conditions, even after sonication. This study underscores the effectiveness of the rGO@PA hybrid membranes for wastewater treatment applications, offering a robust, cost-effective solution for environmental remediation applications.

## Abstract 2

Graphene oxide (GO)-based membranes have gained significant attention for various separation applications over the last decade.??>

## Graphical abstract

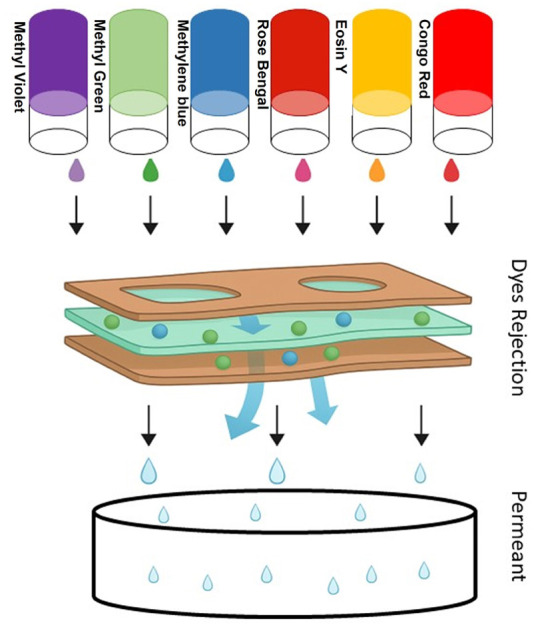

## 1. Introduction

Rapid development in a number of industries, such as electroplating, mining, petrochemicals, textile manufacturing, cosmetics, pharmaceuticals, plastics, battery fabrication, and the paper and pulp industries, have led to the release of a variety of pollutants into water sources without adequate treatment.^1^ Among these pollutants, dyes and colored compounds are the most common pollutants discharged from different industries.^2^ Many of the dyes are poisonous, carcinogenic, mutagenic, and tardily biodegradable organic compounds.^3^ The presence of these dyes in water is thus one of the most significant environmental issues. To date, more than 100 000 dyes are commercially available worldwide, with annual production surpassing 700 000 tons.^4^ Currently, these dyes can be treated physically, chemically, or biologically.^5^ In the biological methods, algae, bacteria, and enzymes are the microorganisms that can be used to degrade dye molecules into less toxic byproducts,^6^ thus offering eco-friendly options. But, due to the complex structure and environmental stability of various organic dyes, such methods are not feasible. On the other hand, chemical methods, such as catalytic ozonation, electrochemical degradation, photocatalytic oxidation, and sonocatalysis, are used for the treatment of these dyes.^7–11^ However, such methods require large quantities of chemicals, high energy, and complex equipment. In contrast, physical methods, including adsorption (using activated carbon, *etc.*), membrane separation, ion exchange and irradiation, are cost-effective and easy to use for the treatment of toxic dyes.^12–14^

Among these methods, membrane filtration has garnered significant attention as a promising approach for dye separation, water purification, and wastewater treatment.^11,15–17^ Membrane technologies, such as microfiltration, ultrafiltration, and reverse osmosis, have the advantage of providing the selective separation of contaminants, including dyes, heavy metals, and salt ions, from wastewater based on size, charge, and other chemical properties.^18–20^ These technologies also offer the benefit of compact systems, ease of operation, and the ability to treat large volumes of wastewater with relatively low energy consumption. Despite the potential of membrane filtration, the technology still faces several challenges that hinder its widespread applications, particularly in the treatment of wastewater contaminated with stable dyes and compounds. One of the main challenges is membrane fouling, which occurs when contaminants, such as organic matter, microorganisms, and metal ions, accumulate on the surface of the membrane, blocking the pores and reducing its performance. This fouling leads to increased resistance to filtration, requiring more frequent cleaning and reducing the operational lifespan of the membrane. Furthermore, the selectivity, permeability, and stability of membranes remain key factors in determining their efficiency for wastewater treatment.^21,22^ As a result, there is a growing need for innovative membrane materials that can overcome these challenges and improve the overall performance of wastewater filtration systems.

Recently, graphene oxide (GO), a two-dimensional material composed of a single layer of carbon atoms with various oxygen-containing functional groups, has attracted considerable interest for use in membrane-based filtration systems.^17,23–26^ GO is a promising material due to its unique combination of high surface area, tunable pore size, and excellent interaction with metal ions, which makes it ideal for separating contaminants from water.^27,28^ The functional groups on the surface of GO, including hydroxyl, epoxide, and carboxyl groups, allow for strong interactions with metal ions, enabling the formation of metal–GO complexes. This interaction is particularly beneficial for removing heavy metals from wastewater, as it allows selective adsorption and separation of metal ions. In addition to its high surface area and functionalization, GO is also known for its ultrathin structure.^29–31^ A monolayer of GO is only about 0.7–1.2 nm thick ^32^ which provides an ideal platform for filtration processes that require fine separations, such as the removal of small ions and organic molecules. Furthermore, the high hydrophilicity of GO helps improve water permeance and desalination properties ^33^ while its antibacterial properties provide an added advantage by reducing the risk of membrane fouling.^34^ However, despite these promising features, GO-based membranes still face limitations, including swelling of the GO sheets when exposed to water, which increases the interlayer distance and reduces membrane stability.^35^ The hydration of GO layers also negatively affects the selectivity and overall performance of the membrane. To address the limitations, reduced GO (rGO) has been explored as an alternative material for membrane separation. The rGO is obtained by chemically reducing GO, which removes some of the oxygenated functional groups, resulting in a material with enhanced chemical stability, improved mechanical properties, and a more controlled pore structure.^36^ The reduction of GO to rGO can help minimize swelling and improve the structural integrity of the membrane, making it more suitable for long-term use in wastewater treatment applications.^37^ However, the process of reducing GO to rGO must be carefully controlled, as the degree of reduction significantly affects the material's properties. Several reduction methods, including thermal reduction, electrochemical reduction, and chemical reduction, have been investigated to optimize the degree of reduction and ensure the desired performance characteristics.^38–40^ While these methods are effective, they often involve high costs and complex setups, and many of the commonly used reducing agents, such as hydrazine, are toxic and pose safety concerns. As a result, the development of non-toxic, cost-effective reducing agents is essential to enhance the performance of rGO-based membranes while ensuring their safety and environmental friendliness. To further enhance the performance of GO-based membranes, a novel approach has been developed by combining GO with aluminum-based materials, specifically potassium aluminum sulfate, to form a composite membrane. This composite material, referred to as GO@Al, is fabricated using a simple vacuum filtration method, which allows for the efficient stacking of GO and Al-MOF nanosheets into a lamellar structure.^41^ The Al-MOF component plays a critical role in improving the stability and performance of the membrane by coordinating with the oxygenated functional groups on the GO sheets. This coordination enhances the structural integrity of the membrane and reduces the swelling effect typically observed with pure GO membranes. Additionally, the high porosity of the Al-MOF nanosheets increases the available surface area for filtration, which enhances the membrane's permeability. The hydrophilic nature of the Al-MOF material also improves water transport through the membrane, reducing filtration resistance and allowing more efficient filtration of wastewater. By combining the strengths of both GO and Al-MOF, the GO@Al composite membrane demonstrates superior performance in removing a wide range of contaminants, including heavy metals, dyes, and salts, from wastewater.

In this work, rGO@PA composite membranes were fabricated using various loading ratios of potash alum and demonstrated exceptional performance in the removal of dyes from contaminated water. The membrane shows excellent dye rejection for anionic dyes, *e.g.*, Congo Red, Eosin Y, and Rose Bengal, achieving rejections up to 99.9%, 99.8%, and 99.2%, respectively. Besides these, the membrane also shows great rejection for cationic dyes, such as 99.0%, 99.6%, and 99.2% for methylene blue, methyl green, and methyl violet, respectively. The high rejection efficiency is attributed to strong electrostatic interactions between the negatively charged dyes and the membrane surface, which prevents the dyes from passing through the membrane. Further, the rGO@PA composite membrane also maintains its performance for over 15 days under aqueous conditions, even after sonication, highlighting its long-term stability.

## 2. Materials and methods

### 2.1. Materials

The chemicals used in the synthesis of the GO nanosheets, namely, graphite flakes (325 mesh particles (50–70%), sulfuric acid (98% H_2_SO_4_), sodium nitrate (ACS reagent, ≥99.0%), and potassium permanganate (97%)), were obtained from Sigma-Aldrich. Potash alum (ACS reagent, ≥98%) was obtained from Sigma-Aldrich and was used for the modification of the GO nanosheets. Nylon filter membranes (Sigma-Aldrich, China) with a pore size of 0.45 µm were used.

### 2.2. Preparation of GO sheets

The GO was prepared according to the reported method.^42^ First, 4.0 g of graphite (325 mesh, Sigma Aldrich Co., Ltd) was mixed with 190 mL of concentrated H_2_SO_4_, and 3.5 g of NaNO_3_ was added with continuous stirring in an ice bath. Then, 18.0 g of KMnO_4_ was slowly added to the above mixture over 15 min, and the temperature was maintained below 20 °C for 2 h. The mixture was then stirred at room temperature for 3 h. Then, 280.0 mL of DI water was added at this stage to avoid the risk of explosion or overheating. Further, 780.0 mL of DI water and 5.0 mL of 30% H_2_O_2_ were added to the mixture to obtain a graphite oxide suspension. The obtained product was washed repeatedly with aqueous HCl solution (3%) and dialyzed for 6 days to eliminate contamination. Then, to exfoliate the graphite oxide into a graphene oxide suspension, tip sonication (135 W, 60 min) was used. Finally, the mixture was centrifuged at 6000 rpm for 40 min to eliminate small pieces and thick multilayered flakes, respectively. The as-obtained GO dispersion was used for further experiments after drying in a vacuum dryer.

### 2.3. Preparation of rGO@PA composite membranes

**Scheme 1:**
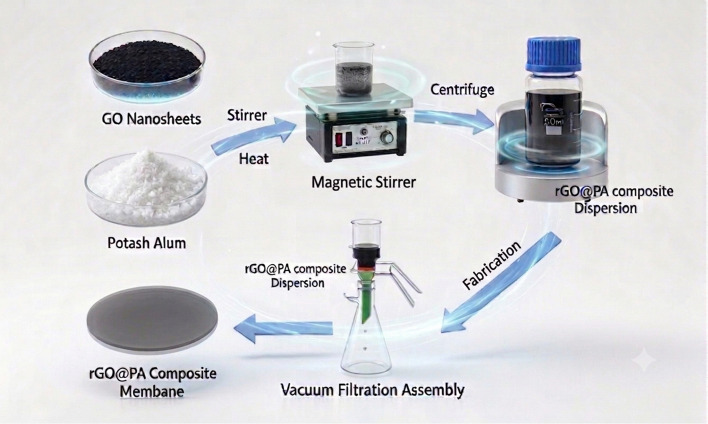
Fabrication of the rGO@PA composite membranes using the vacuum filtration method.

The rGO@PA composite solution was prepared by mixing 0.2 g of GO sheets into 100 mL of DI water, followed by sonication for 1 h to exfoliate the GO sheets (Scheme 1). Then, 6.3 g of potash alum was added with continuous stirring for 2 h, and the pH of the solution was adjusted by adding sodium hydroxide solution with continuous stirring for 16 h to give a dispersion of the rGO@PA composite. The as-prepared rGO@PA composite was used for further fabrication of membranes by using polyethersulfone support (0.45 µm) through vacuum filtration assembly. The loading ratio of PA was adjusted to control the loading ratio of membranes.

### 2.4. Characterization

Scanning electron microscopy (SEM, Nova Nano SEM430) was performed to obtain surface and cross-sectional images of the GO-based membranes. X-ray diffraction (XRD) spectra were recorded using Cu Kα radiation (*λ* = 0.154 nm, D-MAX/2400) to obtain the structural information. X-ray photoelectron spectroscopy (XPS, ESCALAB 250Xi) was used to determine the degree of reduction of the materials and membranes. A Fourier-transform infrared spectrometer (FTIR, Nicolet 6700) was used to analyze the functional groups in the composite. A Bruker DekaXT Profiler (Germany) was used to measure the thicknesses of the membranes. A Mettler Toledo M400 and a Thermo Scientific iCE 3300 were used to measure the rejection of heavy metals and ions. A UV-Visible spectrometer (Carry 40, Varian) was used to measure the concentrations of the dye solution before and after filtration.

### 2.5. Permeation test

The water permeance and dye rejection of the GO-based membranes were tested using a vacuum filtration assembly at room temperature and 0.1 bar pressure. Dye rejection (*R*) was calculated as follows:

1 *R* (%) = 100 × (1 − *C*_P_/*C*_f_),

where *C*_p_ is the dye concentration in the permeate and *C*_f_ is the dye concentration in the feed solution.

Eqn (2) was used to measure the water permeance (*J*) of the membrane (measured in L m^−2^ h^−1^ bar^−1^). The permeance was calculated as follows, with *V* (L) being the volume of water permeated, *A* (cm^2^) the effective membrane area, Δ*t* (h) the time interval for permeation, *P* (1.0 bar) the applied pressure difference across the membrane:2
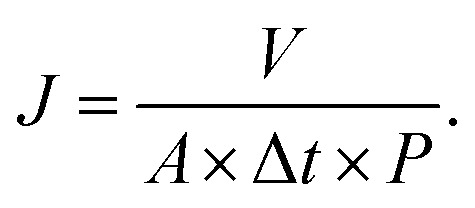


### 2.6. Stability performance

To assess membrane stability, membranes were cut into 1.5 × 1.5 cm^2^ pieces. Each piece was then immersed in solutions of different pH values: acidic (HCl, pH 2), alkaline (NaOH, pH 12), and neutral (pH 7). The stability of the membranes was monitored over various time intervals.

### 2.7. Degree of swelling

The degree of swelling (*d*) of the GO-based membranes was determined according to eqn (3). First, the membrane was soaked in DI water for 12 hours and then dried at room temperature for another 12 hours to perform additional swelling tests.3
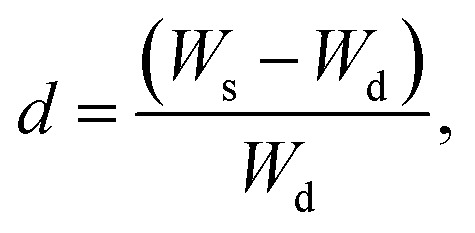
where *W*_s_ and *W*_d_ are the swollen and dry membrane weights, respectively.

## 3. Results and discussions

### 3.1. Physicochemical characterization

#### 3.1.1. Fourier-transform infrared (FTIR) spectroscopy

**Fig. 1:**
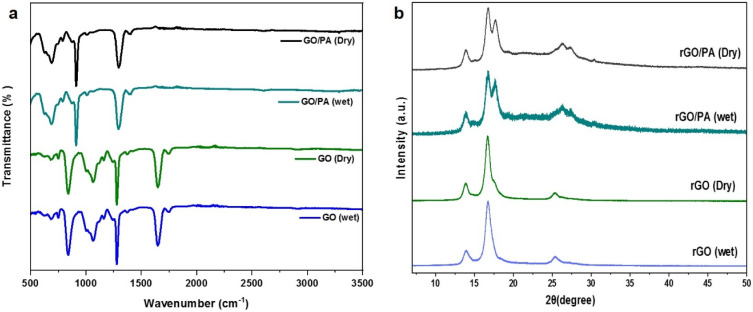
(a) FTIR spectra of the dry and wet pristine GO and rGO@PA composite membranes. (b) XRD patterns of the dry and wet pristine rGO and rGO@PA composite membranes.

The rGO/PA composite-based membranes were characterized by using an FTIR spectrometer, and the spectra were compared with those of pure GO and rGO membranes (Fig. 1a and S1a). The GO spectrum demonstrates the characteristic vibrational patterns resulting from the presence of oxygen-containing functional groups formed during the oxidation of graphite. Fig. 1a showed the very broad and strong band of hydroxyl (O–H) groups at 3400 cm^−1^, which showed the presence of hydroxyl groups (–OH), carboxylic acid groups (–COOH), and especially adsorbed/intercalated water molecules. The band observed near 2920 cm^−1^ can be attributed to C–H stretching vibrations within the sp^2^ carbon domains that remain present. The absorption peak observed around 1720 cm^−1^ corresponds to the C=O stretching vibration of carboxyl and carbonyl groups predominantly located at the edges of the GO sheets. The band around 1620 cm^−1^ is associated with the skeletal vibration of unoxidized graphitic domains (C=C) as well as the bending mode of absorbed water molecules. The peaks observed in the range of approximately 1220 to 1050 cm^−1^ can be attributed to the stretching vibrations of C–O–C (epoxy) and C–O (alkoxy) bonds (Fig. 1a). This indicates that the GO structure exhibits a high level of oxidation. For the rGO (Fig. S1a) and rGO/PA composite (Fig. 1a) membranes, the stretching band for the hydroxyl (O–H) group was not observed, which showed that the GO sheets were reduced and interacted with potash alum. The C=O stretching band at around ∼1720 cm^−1^ exhibits a decrease in intensity in the rGO/PA composite. This indicates that the carboxyl groups of GO and Al^3+^ ions are cooperating or interacting through electrostatic forces. Furthermore, an enhanced characteristic peak was observed within the range of approximately 1100–980 cm^−1^. The overlap of the asymmetric stretching vibrations of sulfate (SO_4_^2−^) groups with the C–O vibrations of the GO peaks distinguishes this phenomenon. Increased absorption in this area is frequently observed in rGO/PA composites and is considered strong evidence for the successful incorporation of sulfate species onto the GO surface. The slight decrease in epoxy-related bands indicates that potash alum species may partially interact with or obscure the basal planes of GO. We have also studied the FTIR spectra of both GO and rGO@PA composite membranes in the wet and dry state (Fig. 1a). The as-prepared membranes showed similar spectra, demonstrating the quality of the membranes. The FTIR studies clearly indicated that potassium alum is not merely physically mixed with GO; it is also chemically and electrostatically bonded through hydrogen bonding and metal oxygen coordination.

#### 3.1.2. X-ray powder diffraction (XRD)

The interlayer distance between the GO sheets of the pure GO, rGO, and rGO@PA composite membranes was characterized by analyzing the XRD patterns (Fig. 1b and S1b). The pure GO membranes showed a considerable diffraction peak at 2*θ* of 11.1° with an interlayer spacing of 0.83 nm, indicating the existence of water molecules and oxygen-containing functions inside the stacking GO nanosheets (Fig. S1b). We also studied the XRD patterns of rGO and rGO@PA composite membranes in both wet and dry states. Fig. 1b shows that the wet rGO membranes exhibit a larger interlayer spacing of 0.883 nm (∼10.0°) compared to the dry rGO membranes, with an interlayer distance of 0.82 nm (∼10.8°), due to the presence of water molecules between the nanosheets. Moreover, we also studied the interlayer spacing of the rGO/PA composite membranes in dry and wet states (Fig. 1b) and compared them with those of pure GO and rGO membranes. The wet rGO/PA composite membrane exhibited two diffraction peaks at 2*θ* of 9.8°, with an interlayer distance of 0.90 nm, and at 23.1°, with an interlayer distance of 0.35 nm, as shown in Fig. 1b. While the dry rGO/PA composite membrane exhibited two diffraction peaks at 2*θ* of 8.42°, with an interlayer distance of 0.84 nm, the weak broad features around 24°–25° (*d* ≈ 0.35 to 0.38 nm) show partial restoration of sp^2^ carbon domains, but the low-angle dominance means reduction is incomplete, as shown in Fig. 1b.

#### 3.1.3. Scanning electron microscope (SEM) and EDS analyses

**Fig. 2:**
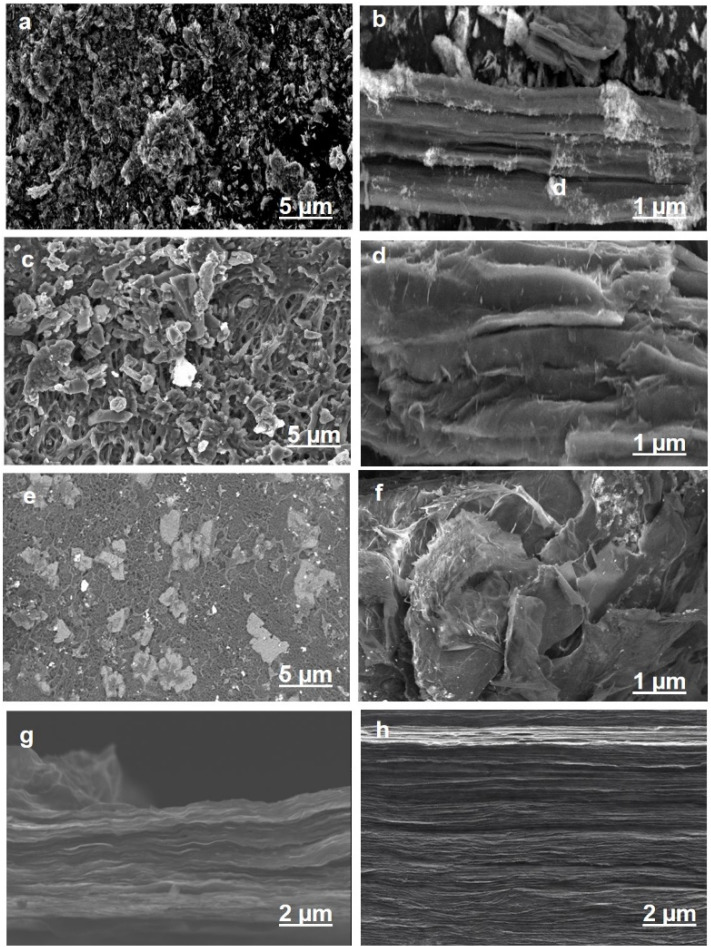
SEM images of the (a and b) GO, (c and d) rGO and (e and f) rGO@PA composite membranes. Cross-sectional images of the (g) rGO and (h) rGO@PA composite membranes.

The surface morphology of pure GO (Fig. 2a and b), rGO (Fig. 2c and d), and rGO/PA composite (Fig. 2e and f) membranes was studied. The pure GO membranes showed rough and flake-like morphology (Fig. 2a and b) similar to rGO membranes (Fig. 2c and d). However, the rGO@PA composite membranes showed a uniform surface morphology (Fig. 2e and f). Furthermore, a large number of plate-like particles (Fig. 2d) are also present in the matrix. We also studied the cross-sectional SEM images of the pure rGO and rGO@PA composite membranes (Fig. 2g and h). The cross-sectional SEM studies showed that the rGO@PA composite membranes have slightly larger interlayer spacing between nanosheets, which might be due to the presence of alum molecules within the nanosheets.

The elemental distribution and composition of the rGO/PA composite membranes were then investigated using energy-dispersive X-ray spectroscopy (EDS) elemental mapping (Fig. S2a–g). The delineated area displays a consistent distribution of carbon and oxygen, corresponding to the GO structure and functional groups that include oxygen. The signals for aluminum, sulfur, and potassium are evenly dispersed, indicating that the potassium aluminum sulfate was successfully integrated into the GO matrix (Fig. S2a–g). The lack of element aggregation indicates that the interfaces interact well, allowing consistent construction of the composite. The EDS spectrum demonstrates the lack of impurities in the elements carbon, oxygen, aluminum, sulfur, and potassium.

#### 3.1.4. Raman studies

**Fig. 3:**
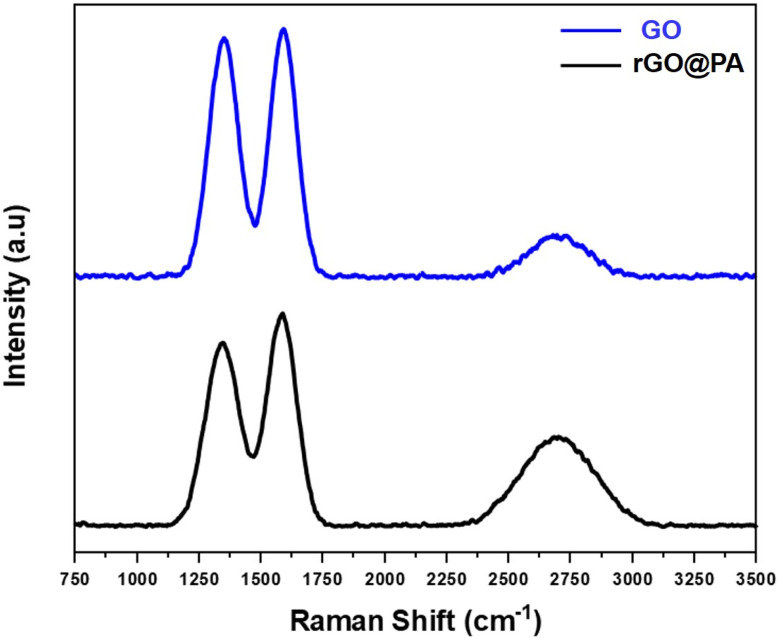
Raman spectra of the pure GO and rGO@PA composite membranes.

Raman studies of pristine GO and rGO/PA composite membranes were carried out (Fig. 3). The GO membrane shows a typical highly oxidized GO spectrum with two strong, broad peaks, an intense D band (∼1350 cm^−1^, disorder/defects from oxygen groups) that is slightly more intense than the G band (∼1600 cm^−1^, sp^2^ carbon stretching). Another weak, broad 2D band appeared at ∼2700 cm^−1^, which indicates a disrupted graphitic structure. Furthermore, no significant peaks were obtained below ∼1200 cm^−1^. However, the Raman spectra of rGO@PA indicated a partial reduction of the GO sheets (Fig. 3); it showed that the D and G bands remain prominent but appear more balanced (lower ID/IG ratio) with slightly sharper features, indicating a partial restoration of the sp^2^ carbon domains and fewer defects after reduction. The 2D band between 2700 and 2800 cm^−1^ is noticeably broader and more intense, confirming the improved graphitic order in rGO. A small sharp peak at ∼1000 cm^−1^ (absent in pure GO) is assigned to the characteristic symmetric SO_4_^2−^ stretch from potash alum (KAl(SO_4_)_2_·12H_2_O), proving successful composite formation. The overall second-order region (∼2500–3200 cm^−1^) is more pronounced. Therefore, the rGO@PA spectrum shows evidence of successful partial reduction (better sp^2^ recovery, stronger 2D) combined with alum integration, while retaining some residual disorder typical of rGO composites.

#### 3.1.5. XPS studies

**Fig. 4:**
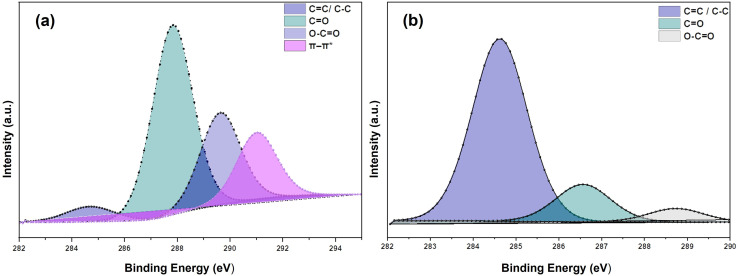
C 1s XPS spectra of the GO (a) and rGO/PA composite (b) membranes.

X-ray photoelectron spectroscopy (XPS) was used to analyze the chemical composition of the GO and rGO@PA composite membranes, as shown in Fig. 4a and b. In Fig. 4a, the pure GO membrane exhibits the typical C 1s spectrum reported for GO membranes ^43^ containing four main components assigned to hydroxyl, epoxy, carbonyl, and carboxyl groups at binding energies of 284.5, 286.0, 288.1, and 291 eV, respectively. After cross-linking GO with potash alum, the intensity of the C–O peak decreased significantly (Fig. 4b). The proportion of C–C/C=C groups increased from 42% to 81%, indicating the restoration of sp^3^/sp^2^-hybridized carbon structures. In the rGO@PA composite membranes, three C 1s peaks appear at 284.5, 286.3, and 288.5 eV, corresponding to C–C, C–O, and C=O bonds, respectively. Fig. 4b shows that the spectrum is largely dominated by C–C/C=C, with only small contributions from oxidized carbon species. This confirms that most oxygen-containing groups were removed, leaving primarily reduced carbon structures. The results were also compared with those for pure alum, as presented in Fig. S3.

### 3.2. Contact angle and stability studies of pristine GO and rGO@PA composite membranes

**Fig. 5:**
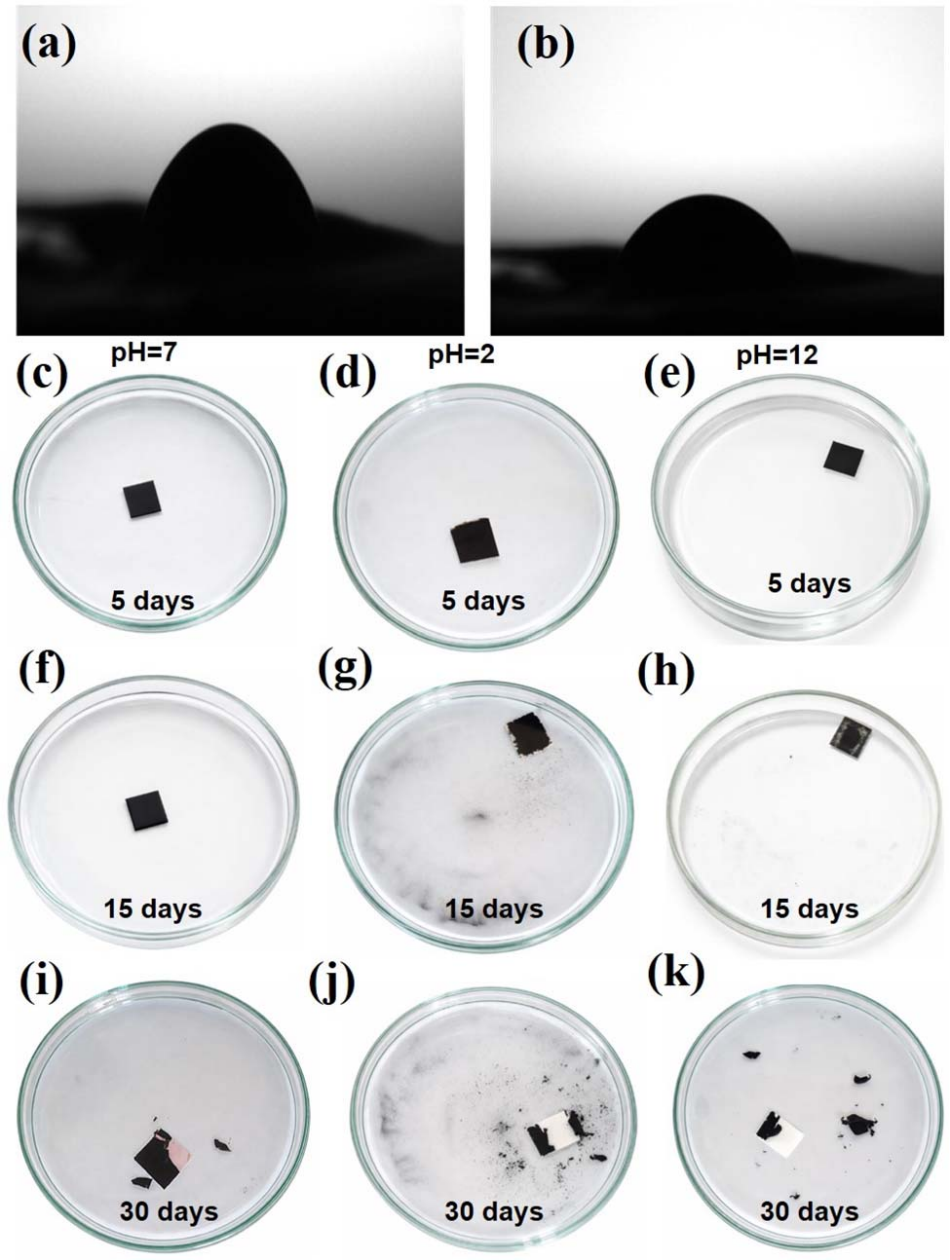
Contact angles of the GO (a) and rGO@PA composite (b) membranes. Stability of the rGO@PA composite membranes under neutral (c, f, and i), acidic (d, g, and j), and basic (e, h, and k) conditions.

The wettability of the pure GO and rGO@PA composite membranes was evaluated using water contact angle measurements, as shown in Fig. 5a and b. The contact angle images indicated that the rGO@PA composite membrane (Fig. 5b) exhibits a more hydrophobic nature, with measured CA angles of approximately 65°, compared to pure GO (Fig. 5a). The hydrophobic characteristics of the membrane are possibly due to the presence of fewer oxygen-containing functional groups (–OH and –COOH) in the rGO sheets and the interaction with aluminum sulfate, which makes a narrow interlayer space between the membranes. The rGO@PA composite membrane remained structurally stable under aqueous conditions, demonstrating excellent potential for long-term use in wastewater treatment. The high hydrophobicity of the rGO@PA composite membrane enhances its separation efficiency for charged solutes, particularly for dye removal and heavy-metal ion rejection. To date, several studies have reported the lower stability of pure GO-based membranes after up to 5 days in water due to the presence of epoxy, hydroxy, carboxyl, and carbonyl groups at the margins and basal plane. Oxygen-containing groups in the GO membrane may have a hydration impact that reduces the GO sheets' capacity to repel each other. In order to improve the water stability of the GO sheets, it is essential that functional groups present in the sheets be controlled. Fig. 5c–k show the stability of rGO@PA composite membranes in neutral (Fig. 5c, f, and i), acidic (Fig. 5d, g, and j), and basic (Fig. 5e, h, and k) media. Our fabricated rGO@PA composite membranes exhibited excellent stability at different pH levels. Fig. 4c illustrates the stability of the as-rGO@PA composite membranes in water (pH 7) for up to 15 days. However, the membranes are also stable for up to 10 days under acidic (pH 2) and basic (pH 12) conditions, as shown in Fig. 5g and h. The membrane is completely delaminated within 30 days. The low stability of these membranes over extended periods is possibly due to the hydration effect of GO nanosheets with water molecules.

### 3.3. The nanofiltration efficiency and water permeance of the membranes

#### 3.3.1. Water permeance of the membranes

**Fig. 6:**
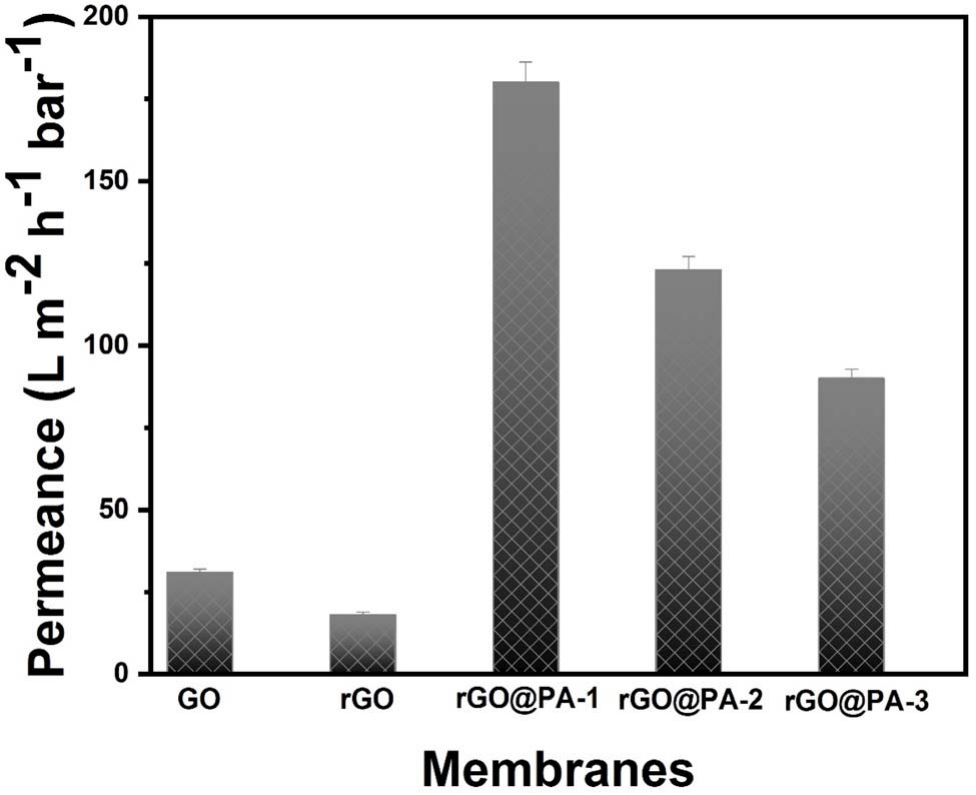
Water permeance performance of the GO, rGO and rGO@PA composite membranes with different loading ratios at a pressure of 1 bar.

Several studies suggested that GO membranes show reduced water permeance due to their tight interlayer spacing. We have also measured the water permeance of the GO membrane, and found that it exhibited a water permeance of 31 ± 1 L m^−2^ h^−1^ bar^−1^ (Fig. 6). We also measured the water permeance of the rGO membrane and found that it showed less water permeance (18 ± 1 L m^−2^ h^−1^ bar^−1^). Furthermore, the water permeances of the rGO@PA composite membranes with different loading ratios (1 : 1, 1 : 2, and 1 : 3) were measured (Fig. 6). The as-prepared rGO@PA composite membranes with different loading ratios (1 : 1, 1 : 2, and 1 : 3) were named rGO@PA-1, rGO@PA-2, and rGO@PA-3, respectively. The rGO@PA-1 composite membrane exhibited a water permeance of 180 ± 2 L m^−2^ h^−1^ bar^−1^, which is five times greater than that of the pure GO membrane. Furthermore, we observed that the water permeance decreased as the loading ratio of potash alum increased. The rGO@PA-3 composite membranes showed low water permeance of up to 90 ± 2 L m^−2^ h^−1^ bar^−1^, which is possibly due to narrower interlayer spacing and reduced GO nanosheets.

#### 3.3.2. Nanofiltration efficiency

**Fig. 7:**
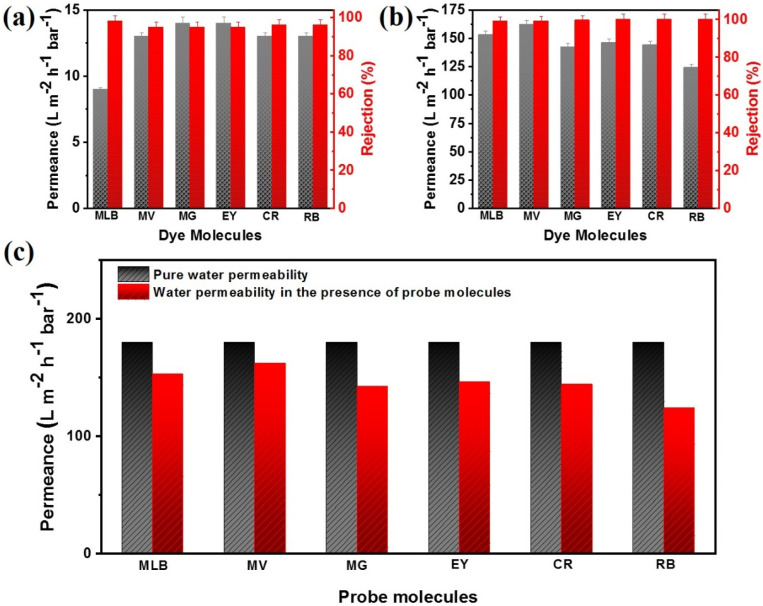
Retention performance of the (a) pure GO and (b) rGO@PA composite membranes using different probe molecules with different charges and sizes (concentration of each dye = 100 ppm). (c) Overall 10–20% decline in water permeance observed for the MLB, MV, MG, EB, CR and RB molecules.

Initially, the rejection efficiency of the pure GO membrane was investigated using MLB, MV, MG, CR, EY, and RB dyes (Table 1). The GO membranes showed high rejection efficiency (98.2% for EB dye) but with reduced permeance. However, such membranes showed very low rejection efficiency, *i.e.*, 95% ± 1%, for the MG, MV, RB, CR, and EY dyes (Fig. 7a). Furthermore, we used several dyes, such as MLB, MG, MV, RB, CR, and EY, with varying masses and sizes to assess the rejection efficiency and molecular cutoff of the as-prepared rGO@PA composite membranes (Fig. 7b). GO nanosheets are good absorbers of dyes and heavy-metal ions due to the presence of functional groups. Therefore, to assess the accuracy of the performance results, we removed the adsorption effect of the GO membranes. In this work, we stabilized the samples for 60 min at low pressure (0.6 bar) to prevent adsorption before the feed and permeate solutions were measured. The feed and permeate concentrations were then analyzed using a UV/Vis spectrophotometer (Fig. S4). Interestingly, the as-prepared rGO@PA composite membrane rejected all the dyes with >99% efficiency (Fig. 7a). This rejection efficiency is several times greater than that reported for other GO membranes (Table S1). This larger rejection and decrease in water permeance are due to the narrow interlayer spacing of the membranes. Thus, the modification of the GO sheets with potash alum results in enhance in separation efficiency due to the presence of multiple functional groups (hydroxyl and amino) and decreased nanosheet spacings. Therefore, the membrane acts as an efficient molecular sieve agent. The rejection efficiency of dyes also depends on their sizes, charges, shapes, and molecular weights. In our study, charge and size were major factors in the high rejection rate, and the results showed that the rGO@PA composite membranes have a highly positively charged surface.

**Table 1:** Filtration efficiency of the pure GO and rGO@PA composite membranes using different dyes (100 ppm)^a^

Feed solution	Molar mass (g mol^−1^)	Pristine GO		rGO@PA composite	
		Rej (%)	Perm (L m^−2^ h^−1^ bar^−1^)	Rej (%)	Perm (L m^−2^ h^−1^ bar^−1^)
MLB	319.8	98.2	9 ± 1	99.0	153 ± 2
MV	393.0	95.1	13 ± 1	99.2	162 ± 2
MG	458.5	95.1	14 ± 1	99.6	142 ± 2
EY	647.8	95.1	14 ± 1	99.8	146 ± 2
CR	696.6	96.1	13 ± 1	99.9	144 ± 2
RB	973.7	95.1	13 ± 1	99.2	124 ± 2

a Abbreviations: CR: Congo red, MLB: methylene blue, EY: Eosin Y, MG: methyl green, RB: Rose Bengal, MG: methylene violet, Rej: rejection, and Perm: permeance.

Therefore, highly positively charged surfaces repel cationic dyes with high efficiency (Fig. 7b). Furthermore, our study also clearly showed that the membranes also exhibit higher rejection of large dye molecules compared to small dye molecules. In addition, molecular sieving agents also play a major role in separation efficiency. The high rejection efficiency of these membranes is possibly due to their absorption, electrostatic interaction, and size exclusion. Furthermore, adsorption plays a vital role; the surface of the rGO@PA composite membrane adsorbs different dye molecules on its surface through electrostatic interactions. Therefore, this process is also responsible for the high rejection efficiency of dyes. Electrostatic interactions are the key component that leads to the good rejection efficiency of our membranes. In addition, we have studied the effect of the loading ratio of the cross-linking reagent (potash alum) on the permeance and separation efficiency of the membranes with respect to the dyes. As we increased the potash alum ratio from 1 : 1 to 1 : 3, the membrane water permeance increased; however, dye rejection decreased to below 60% for all dyes. These findings demonstrate that the rGO@PA composite membrane's pore size is crucial for separation applications. Such an rGO@PA composite membrane exhibits an excellent balance of permeance and rejection, which is many times greater than the reported GO-based NF membranes. However, the permeance is found to be around 10%–20% lower than the pure solvent permeance (Fig. 7c), which may be caused by the solute molecules blocking the nanochannels.

Furthermore, the as-prepared rGO@PA-1 composite membrane was tested for its rejection efficiency to remove CR dye at different feed concentrations (100, 150, 200, 250 and 350 ppm). As the concentration of the feed solution increased, the rejection efficiency of the dye decreased several times. As the concentration of dye increases, more dye molecules accumulate on the membrane surface. This leads to surface oversaturation, meaning the available active sites on the membrane become fully occupied. Once saturation occurs, additional dye molecules cannot be effectively adsorbed or blocked. The rGO@PA-1 composite membrane showed 45% rejection for a 350 ppm feed solution (Table S2). We anticipate that the rGO@PA membranes, with their excellent separation performance, hold strong potential for a wide range of future separation applications.

## 4. Conclusion

In this study, rGO@PA composite membranes were fabricated by modifying GO sheets with potash alum. The as-prepared rGO@PA composite membrane achieved high water permeance of up to 180 ± 10 L m^−2^ h^−1^ bar^−1^ and demonstrated excellent separation of both anionic and cationic dyes as well as excellent dye rejection for anionic dyes, *e.g.*, Congo Red, Eosin Y, and Rose Bengal, achieving rejections of up to 99.9%, 99.8%, and 99.2%, respectively. Besides these, the membrane also shows great rejection for cationic dyes, such as 99.0%, 99.6%, and 99.2% for methylene blue, methyl green, and methyl violet, respectively. In addition, the rGO@PA composite membrane is more stable than pristine GO-based membranes. The membrane remained stable for up to 15 days in neutral solution and 10 days in basic and acidic solutions. Due to the perfect pore size attained by the green approach, our produced membranes offer several advantages over GO and rGO membranes described in the literature. Therefore, such membranes offer an alternative solution for various wastewater treatments and selective separations in the future, after a few modifications.

## Conflicts of interest

With the permission of all the authors, the corresponding author declares no conflicts of interest related to this work.

## Supplementary Material

RA-016-D6RA00858E-s001

## Data Availability

Data will be made available from the corresponding author upon reasonable request. Supplementary information (SI) is available. See DOI: https://doi.org/10.1039/d6ra00858e.
