# Supplementary material for: Eco-friendly reduced graphene oxide@potash alum-based composite membranes for efficient separation of dyes and selective removal of contaminants from wastewater
Source: RSC Adv. 2026 Jul 2;16(34):32527–37. doi: 10.1039/d6ra00858e (PMC13325947; doi:10.1039/d6ra00858e)
Supplement: RA-016-D6RA00858E-s001 [file RA-016-D6RA00858E-s001.pdf]

Supplementary Information (SI) for RSC Advances.
This journal is © The Royal Society of Chemistry 2026

SUPPLEMENTARY MATERIAL

Eco-Friendly Reduced Graphene oxide@Potash Alum-based Composite Membranes for Efficient Separation of Dyes and Selective Removal of Contaminants from Wastewater

Irsa Munwar[1], Akbar Ali[2], Ashiq Hussain Jatoi[3], Khalid Hussain Thebo[4*], Ahmed Nadeem[5]

[1]National Centre of Excellence in Analytical Chemistry, University of Sindh, Jamshoro, Pakistan

[2]MIIT Key Laboratory of Critical Materials Technology for New Energy Conversion and Storage, State Key Laboratory of Urban Water Resource and Environment, School of Chemistry and Chemical Engineering, Harbin Institute of Technology, Harbin 150001, PR China

[3]Department of Chemistry, Shaheed Benazir Bhutto University, Shaheed Benazirabad, Pakistan

[4]Department of Chemistry, Mirpur University of Science & Technology (MUST), Mirpur Pakistan

[5]Department of Pharmacology and Toxicology, College of Pharmacy, King Saud University, Riyadh 11451, Saudi Arabia

*Corresponding Authors Email: khalidthebo@yahoo.com**

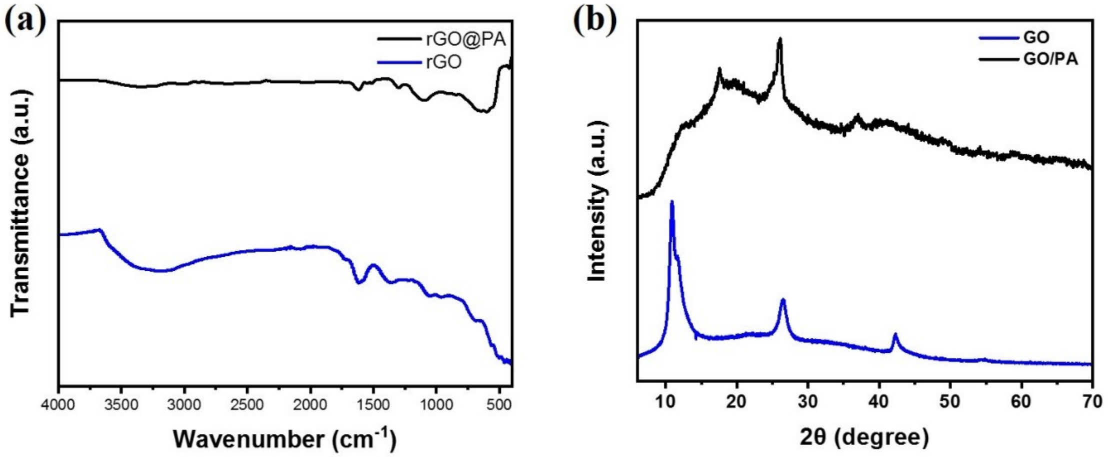

Fig. S1. (a) The FTIR spectra and (b) XRD pattern of pure GO and GO@PA composite membranes, respectively.

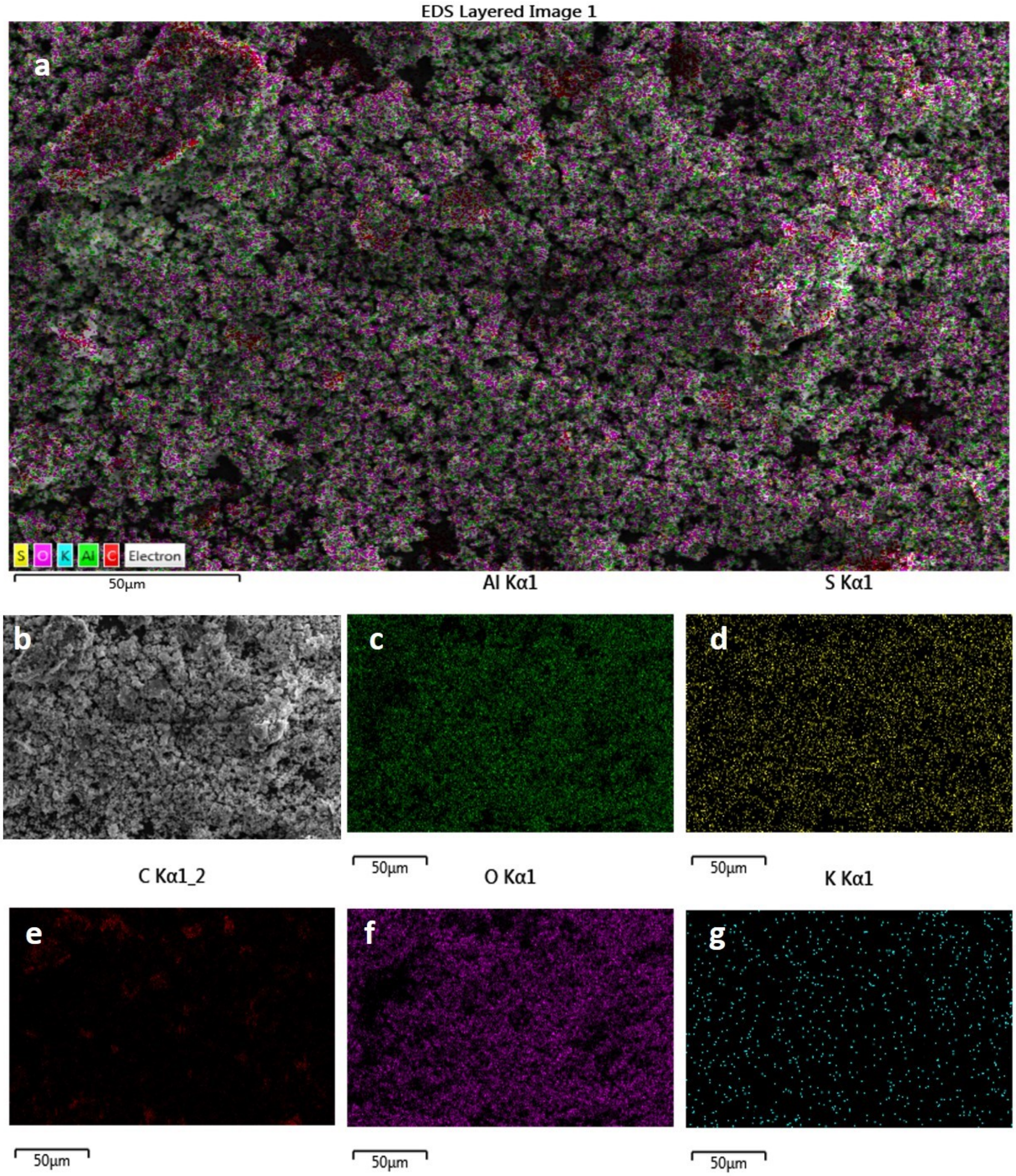

Fig. S2. (a-g) Illustration elemental mapping through EDS, and EDS spectra of the GO/potassium aluminum sulfate composite. The uniform distribution of C, O, Al, S, and K indicates that the composite was synthesized effectively, demonstrating the compatibility of graphene oxide and potassium aluminum sulfate at the interface, respectively.

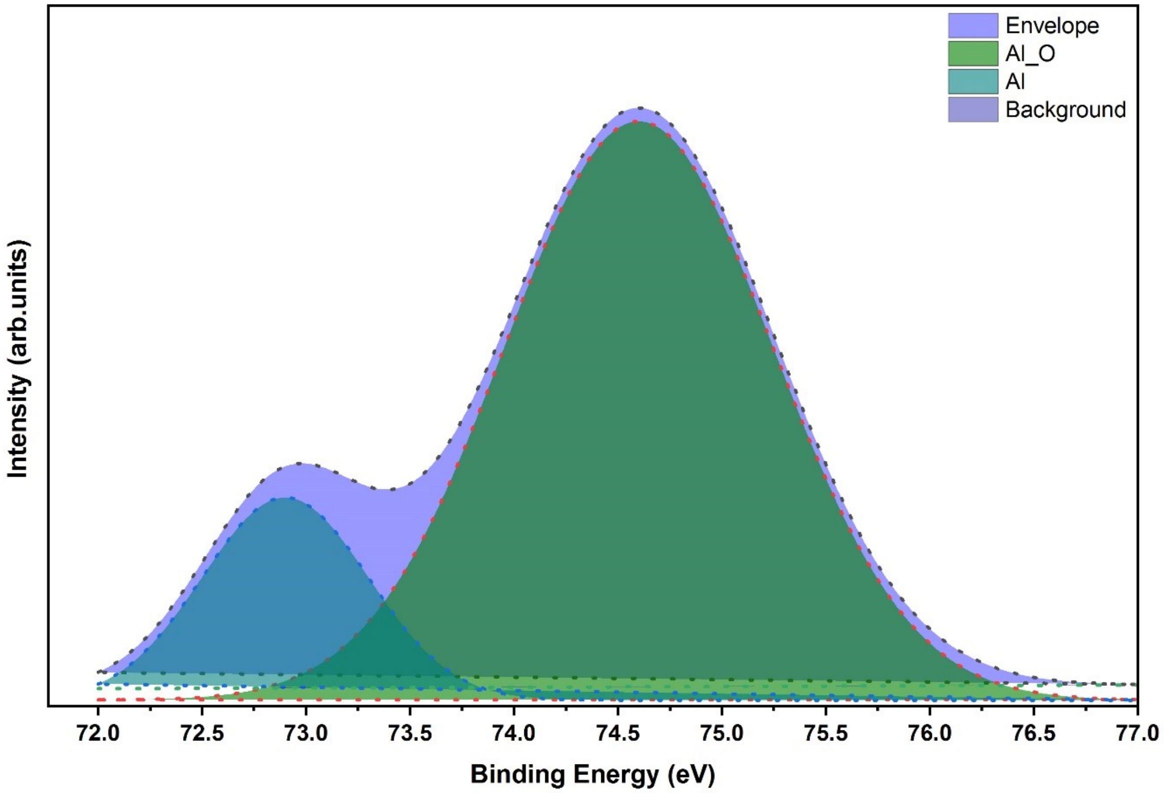

Fig. S3. XPS spectra of pure alum, respectively.

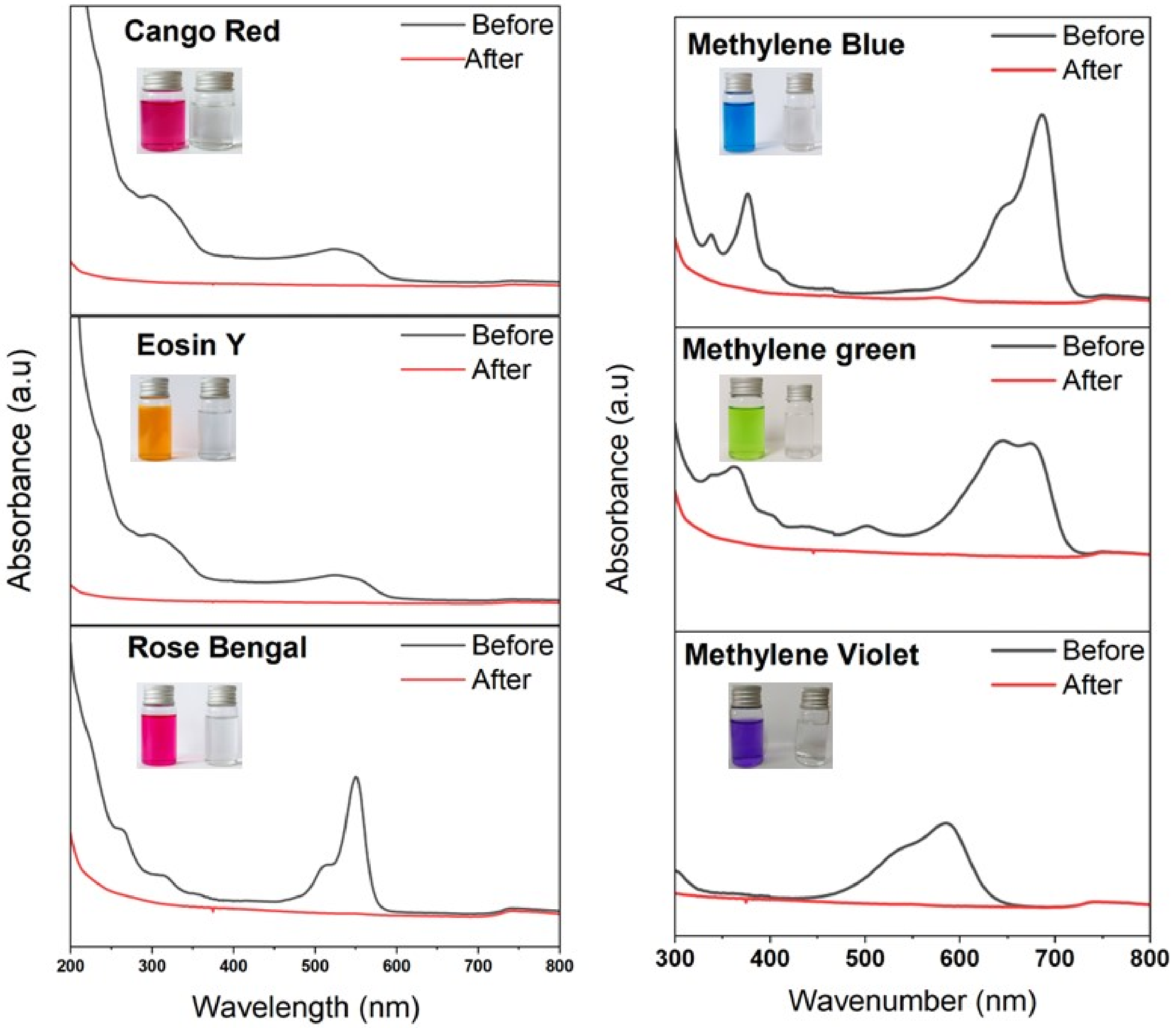

Fig. S4. UV-Vis spectra of the feed, retentate and permeate of (a) CR, (b) MLB, (c) EY, (d) MG, (e) RB, and (f) MV feed solution through rGO@PA composite membrane

Table S1: Dye rejection by graphene-based membranes

Types of Membranes	Fabrication Method	Membrane structure^a	Types of feed / Conc.	Permeance (LMHB)	Rejection (%)	Ref.
rGO/GO composite	Vacuum filtration	-	MB	99.0	201.7	[1]
NSC-GO/PC	Vacuum filtration	l = 2.03 μm	EB (15 mM)	695	83.5	[2]
			RB (1 mM)	279	87	
ZnO@rGO		-	MB	225	98	[3]
Base-refluxing reduced GO/PVDF	Vacuum filtration	l = 22	MB (0.02 mM)	21.8	99.2	[4]
TMC cross-linked GO/PSF	Layer-by-layer	l = 5–50 layers	MB (7.5 mg L)	8–27.6	46–66	[5]
nAg-rGO/n-MIL	vacuum-assisted and pressure-assisted filtration techniques	-	MB (10 ppm)	21.7	97.5	[6]
Porous GO/PC	Vacuum filtration	l = 1 μm	EB (10 mM)	191 -	99	[7]
GO/Ag NPs functionalised membranes	Vacuum filtration	~385 ± 10 nm	MLB	287 ± 10	99 ± 1	[8]
GO/FLG/ deoxycholate membrane	Spray-coating	T=26–33 nm	RB	- -	8	[9]

Abbreviation:^{a}*d*: pore size; *l*: membrane thickness; GO: graphene oxide; NSC: Nanostrand channeled; TMC: 1,3,5-benzenetricarbonyl trichloride; SWCNT: Single walled CNT; AAO: Anodic Aluminum Oxide; PVDF: polyvinylidene difluoride; PAN: polyacrylonitrile; PAH: poly(allylaminehydrochloride); RB: Rhodamine B; MB: Methyl Blue; EB: Evans Blue; FLG: few layered graphene.

Table S2: The rejection efficiency of rGO@PA composite membranes using different concentration of feed solutions (CR Dye)

**Feed solution**	**rGO@PA composite**	
	**Rej (%)**	**Perm ($L\ m^{-2}\ h^{-1}\ bar^{-1}$)**
100	99.9	153±2
150	97.3	157±2
200	82.6	160±2
250	67.0	164±2
350	45.0	175±2

References

1. Liang, S., et al., *Ultrahigh water permeance of a composite reduced graphene oxide/graphene oxide membrane for efficient rejection of dyes.* New Journal of Chemistry, 2024. **48**(41): p. 17706-17710.
2. Huang, H., et al., *Ultrafast viscous water flow through nanostrand-channelled graphene oxide membranes.* Nature Communications, 2013. **4**: p. 2979.
3. Zhang, W., et al., *General synthesis of ultrafine metal oxide/reduced graphene oxide nanocomposites for ultrahigh-flux nanofiltration membrane.* Nature Communications, 2022. **13**(1): p. 471.
4. Han, Y., Z. Xu, and C. Gao, *Ultrathin Graphene Nanofiltration Membrane for Water Purification.* Advanced Functional Materials, 2013. **23**(29): p. 3693-3700.
5. Hu, M. and B. Mi, *Enabling Graphene Oxide Nanosheets as Water Separation Membranes.* Environmental Science & Technology, 2013. **47**(8): p. 3715-3723.
6. Song, S.H., et al., *Silver nanoparticle-decorated reduced graphene oxide/ nanocrystalline titanium metal-organic frameworks composite membranes with enhanced nanofiltration performance and photocatalytic ability.* Desalination and Water Treatment, 2024. **320**: p. 100836.
7. Ying, Y., et al., *In-plane mesoporous graphene oxide nanosheet assembled membranes for molecular separation.* RSC Advances, 2014. **4**(41): p. 21425-21428.
8. Sharif, S., et al., *Functionalised graphene oxide-based nanofiltration membranes with enhanced molecular separation performance.* Materials Research Innovations, 2021: p. 1-9.
9. Morelos-Gomez, A., et al., *Effective NaCl and dye rejection of hybrid graphene oxide/graphene layered membranes.* Nature Nanotechnology, 2017. **12**: p. 1083.
